# Brief learning induces a memory bias for arousing-negative words: an fMRI study in high and low trait anxious persons

**DOI:** 10.3389/fpsyg.2015.01226

**Published:** 2015-08-21

**Authors:** Annuschka S. Eden, Vera Dehmelt, Matthias Bischoff, Pienie Zwitserlood, Harald Kugel, Kati Keuper, Peter Zwanzger, Christian Dobel

**Affiliations:** ^1^Institute of Biomagnetism and Biosignalanalysis, University Hospital of MünsterMünster, Germany; ^2^Institute of Psychology, University of MünsterMünster, Germany; ^3^Institute of Sport and Exercise Sciences, University of MünsterMünster, Germany; ^4^Department of Psycholinguistics and Cognitive Neurosciences, Institute of Psychology, University of MünsterMünster, Germany; ^5^Department of Clinical Radiology, University of MünsterMünster, Germany; ^6^University of Hong KongHong Kong, Hong Kong; ^7^kbo-Inn-Salzach Clinic, Academic Hospital of Psychiatry, Psychotheray and NeurologyWasserburg am Inn, Germany; ^8^Department of Psychology, University of BielefeldBielefeld, Germany; ^9^Department of Otolaryngology, Jena University HospitalJena, Germany

**Keywords:** trait anxiety, fMRI, emotions, memory bias, consolidation, statistical word-learning, amygdala

## Abstract

Persons suffering from anxiety disorders display facilitated processing of arousing and negative stimuli, such as negative words. This memory bias is reflected in better recall and increased amygdala activity in response to such stimuli. However, individual learning histories were not considered in most studies, a concern that we meet here. Thirty-four female persons (half with high-, half with low trait anxiety) participated in a criterion-based associative word-learning paradigm, in which neutral pseudowords were paired with aversive or neutral pictures, which should lead to a valence change for the negatively paired pseudowords. After learning, pseudowords were tested with fMRI to investigate differential brain activation of the amygdala evoked by the newly acquired valence. Explicit and implicit memory was assessed directly after training and in three follow-ups at 4-day intervals. The behavioral results demonstrate that associative word-learning leads to an explicit (but no implicit) memory bias for negatively linked pseudowords, relative to neutral ones, which confirms earlier studies. Bilateral amygdala activation underlines the behavioral effect: Higher trait anxiety is correlated with stronger amygdala activation for negatively linked pseudowords than for neutrally linked ones. Most interestingly, this effect is also present for negatively paired pseudowords that participants could not remember well. Moreover, neutrally paired pseudowords evoked higher amygdala reactivity than completely novel ones in highly anxious persons, which can be taken as evidence for generalization. These findings demonstrate that few word-learning trials generate a memory bias for emotional stimuli, indexed both behaviorally and neurophysiologically. Importantly, the typical memory bias for emotional stimuli and the generalization to neutral ones is larger in high anxious persons.

## Introduction

Emotionally arousing situations and stimuli are processed preferentially. This has been shown by a vast body of studies, with methods ranging from simple behavioral measures to state-of-the-art imaging techniques (e.g., Junghöfer et al., 2001; Koster et al., 2006; Laeger et al., 2012; Eden et al., 2014; Weierich and Treat, 2015; for a meta-analysis, see Bar-Haim et al., 2007; for a review, see Cisler and Koster, 2010). Preferential processing leads to a memory bias, apparent in enhanced memory for emotional as compared to neutral stimuli. This has been shown for many stimulus types, such as pictures (Bradley et al., 1992; Schupp et al., 2004a; Touryan et al., 2007; Yegiyan and Yonelinas, 2011), faces (Schupp et al., 2004b), scenes (Heuer and Reisberg, 1990), gestures (Flaisch et al., 2011), and words (Kissler et al., 2007, 2009; Herbert et al., 2008; Scott et al., 2009; Laeger et al., 2012; Keuper et al., 2013, 2014; Eden et al., 2014). The memory bias, especially for stimuli that are negative and arousing, seems more prominent in persons suffering from an anxiety disorder (Calvo et al., 1994; Friedman et al., 2000; Dalgleish et al., 2003; Eysenck et al., 2007) or from a subclinically anxious personality (Norton et al., 1988; McCabe, 1999; Russo et al., 2006; Mühlberger et al., 2009; Eden et al., 2014). The latter group exhibits high levels of trait anxiety, does not meet the criteria for an anxiety disorder, but is prone to develop one (e.g., McCabe, 1999; Russo et al., 2006; Mitte, 2008; Waldhauser et al., 2011). Increased preferential processing of negative stimuli, difficult disengagement from such stimuli, attentional avoidance, and their underlying mechanisms explain part of the anxiety disorders’ etiology (e.g., Eden et al., 2015; for a review, see Cisler and Koster, 2010). Thus, for a better understanding of this large group of disorders, and for the improvement of therapeutic treatments, it is crucial to understand the mechanisms that underlie the acquisition and processing of arousing-negative stimuli. To this aim, we compare persons with high and low (subclinical) anxiety in an associative learning paradigm, using behavioral and neuroimaging measures.

Learning and memory have explicit and implicit components that are indexed by different measures and methods. Explicit memory involves conscious recollection of previous experience and information (semantic, episodic, and autobiographic). Memory is typically measured by explicit recall or recognition of learned material. In contrast, memory access remains unaware in implicit memory, as is the case for procedural information used in tie-knotting or bike-riding. Implicit memory reveals itself through priming, measured in tasks such as word-fragment completion (e.g., Eysenck and Byrne, 1994), or valence judgment of recently acquired words – when explicit knowledge about their meaning is absent. Implicit and explicit memories differ, and have different neurobiological correlates (e.g., Starr and Phillips, 1970; Cohen and Squire, 1980; Nissen and Bullemer, 1987; Gabrieli et al., 1995; Rugg et al., 1998). The memory bias for emotional stimuli is more reliable in explicit than in implicit measures (Eysenck and Byrne, 1994; Russo et al., 2006; for a review, see Mitte, 2008). Mitte (2008) showed that (trait) anxiety has an impact on explicit measures such as recall, but not on recognition. The existence of an implicit memory bias for emotional information is still under debate. While some provide support for its existence (e.g., Williams et al., 1988, 1996, 1997; Eysenck and Byrne, 1994), others could not replicate their findings (McCabe, 1999; Russo et al., 1999). Our results might contribute to this controversy.

The memory bias can be detected with neuroimaging measures such as functional magnetic resonance imaging (fMRI), which is sensitive enough to even reveal emotional responses to items that cannot be recalled explicitly (Dannlowski et al., 2007b). As neural correlates of emotion processing many fMRI studies have focused on limbic structures, particularly the amygdalae (e.g., Davis and Whalen, 2001; Phelps and LeDoux, 2005). It has been repeatedly demonstrated that the amygdala is hyperactive in response to threatening and negative emotional stimuli. This hyperactivity is evident for faces (e.g., Birbaumer et al., 1998; Sheline et al., 2001; Phan et al., 2006; Dannlowski et al., 2007a,b; Evans et al., 2008; Hall et al., 2014), scenes (e.g., Kryklywy et al., 2013; Frank and Sabatinelli, 2014; Radua et al., 2014), and words (e.g., Isenberg et al., 1999; Strange et al., 2000; Tabert et al., 2001; Hamann and Mao, 2002; Cunningham et al., 2004; Herbert et al., 2011; Kanske and Kotz, 2011; Straube et al., 2011; Laeger et al., 2012; Hoffmann et al., 2015), and is assumed to explain part of the pathogenesis and the maintenance of anxiety (disorders; e.g., Etkin and Wager, 2007; Etkin et al., 2010; Shin and Liberzon, 2010; Xu et al., 2013; Binelli et al., 2014; but see Dresler et al., 2013 for an alternative view). Given the ongoing debate about the neurobiological basis of (trait) anxiety, it is crucial to incorporate imaging measurements into the study design, especially when stimuli are presented below threshold, or trained in a shallow learning paradigm that does not guarantee robust explicit access. The latter is the case in our study, in which persons with high or low levels of anxiety should learn to associate meaning to pseudowords with only few learning instances. Pseudowords were either paired with neutral or with emotionally negative content, and tested with explicit as well as implicit memory measures. We investigated the memory bias for novel emotional (and neutral) words in persons with subclinically high and low trait anxiety. A criterion-based approach during learning allowed for distinguishing between novel words whose meaning could be accessed explicitly, and words that were learned less well.

Despite the many studies on emotion, our approach allows to address some important issues. First, we investigate memory bias for neutral stimuli that gain their emotional connotation via associative learning (similarly as in Eden et al., 2014). These stimuli possess no specific innate or learned valence (in contrast to a picture of an attacking tiger, or of a weapon). With meaningless and association-free pseudowords, we aim at better control of the individual learning histories and the depth of encoding. Second, we use explicit and implicit memory measures to represent these differing aspects of memory. Third, we applied a criterion-based learning approach, ensuring that participants learned the same amount of word-picture pairs, and were presented with equal sets of explicitly learned and less well-learned items in the fMRI measurement, independent of their learning history. Fourth, we included an fMRI measurement into our design to analyze the amygdala’s response to the newly acquired and to completely novel pseudowords.

In the following, we describe the rationale and the background of our learning design in more detail. With a statistical learning paradigm (e.g., Saffran et al., 1996; Breitenstein and Knecht, 2002; Saffran, 2002; Breitenstein et al., 2007; Cunillera et al., 2010; Eden et al., 2014; Laeger et al., 2014a), neutral pseudowords were paired with either arousing-negative or neutral pictures. This paradigm implements an increased conjoint probability of two events (“correct pairings”) throughout the training, compared to two events with a random contingency (“incorrect pairings”). Participants extract relevant information without receiving feedback and without knowing the underlying learning principle – a shallow type of learning. By repeated presentation of stimulus combinations, highly robust long-term learning is possible. The more repetitions, the stronger the associations between the stimuli, which results in a typical learning curve. Hebbian cell assemblies probably provide the neural bases of these processes (Pulvermüller, 1999). The paradigm has some ecological validity and can be taken as a model for language acquisition in children and adults (Dobel et al., 2009, 2010). It also allows the investigation of explicit and implicit effects of shallow learning (as investigated similarly in our prior study, Eden et al., 2014).

Two groups of participants, one high and one low in trait anxiety, were tested. Various measurements were used to evaluate and analyze potentially biased memories: a valence rating, a cued-recall test and an fMRI assessment. The behavioral tests were presented directly after, 4, 8, and 12 days after learning, to assess effects of consolidation and forgetting. The valence rating entailed a spontaneous evaluation of the pseudowords’ (gained) valence. Valence ratings do not require explicit memory and can thus tap into implicit memory, indexing the valence transferred from pictures to formerly neutral pseudowords. We chose the valence rating because it has been effectively applied after only a few learning instances, when explicit measures are not yet sensitive to learning (e.g., Steinberg et al., 2012; Eden et al., 2014). The cued-recall test measured explicit knowledge about the acquired meaning of the pseudowords. It is comparable to a vocabulary test.

In Eden et al. (2014), we used a very similar design to investigate learning of emotional words in persons with high anxiety. We obtained very little evidence for a memory bias during learning, but explicit and implicit measures revealed a bias after learning. High-anxious persons displayed a stronger memory bias than low-anxious individuals. In fact, they even judged neutrally paired words as negative when their meaning could not explicitly be recalled. We took this effect as evidence for generalization (Eden et al., 2014). Given these findings and the current literature, we expected for the current study enhanced memory effects (i.e., memory bias) for aversive stimulus material immediately after learning and a stabilization of this effect after a time delay allowing consolidation. In line with Mitte (2008), we predicted a stronger bias in explicit cued-recall and fMRI measurements than in (implicit) valence ratings. We also expected a more pronounced bias in high-anxious individuals, who should also show a generalization effect, with increased emotional valence for neutrally paired pseudowords after training. We included completely novel pseudowords for particular fMRI contrasts (see below).

In sum, we instantiated shallow learning via the combination of pseudowords with arousing-negative or neutral picture content, which should nevertheless lead to a memory bias. Next to behavioral effects we focused on neurophysiological consequences of learning, as indexed by amygdalar activity. For this, we contrasted the following conditions:

(a) Explicitly learned pseudowords vs. novel pseudowords (contrast 1); less well-learned pseudowords vs. novel pseudowords (contrast 2). These comparisons inform about general learning effects for new word forms, even if they were not learned well explicitly.
(b) Arousing pseudowords vs. neutral pseudowords (contrast 3). This contrast displays word-affect effects.
(c) Explicitly learned arousing pseudowords vs. less well-learned arousing pseudowords (contrast 4). This comparison informs about similarities or differences between explicitly learned vs. less well-learned emotionally arousing-negative stimuli.
(d) Less well-learned arousing-negative pseudowords vs. less well-learned neutral pseudowords (contrast 5); less well-learned neutral pseudowords vs. novel pseudowords (contrast 6). Less well-learned arousing-negative vs. novel pseudowords (contrast 7). These contrasts investigate effects of word-affect when words are not learned and remembered well.
(e) Neutral pseudowords vs. novel pseudowords (contrast 8), to assess generalization effects reported earlier (Eden et al., 2014). This contrast tested additionally whether pseudowords paired with neutral meaning evoke higher activity than completely novel pseudowords.

In a second step, we tested whether effects are mediated by trait anxiety. This was done by entering trait anxiety as covariate.

## Materials and Methods

### Ethics Statement

All procedures were cleared by the ethical review board of the Ärztekammer Westfalen-Lippe and subjects gave informed consent to participate. All clinical investigation was conducted according to the principles of the Declaration of Helsinki.

### Participants

The Spielberger State Trait Anxiety Inventory (Spielberger et al., 1983) was completed via online survey by 310 non-clinical participants. On the basis of individual scores, 17 participants scoring thirty or below in the trait-anxiety inventory (range: 20–80) were assigned to the low-anxiety group (mean trait score = 27.76, SD = 4.15; mean age 26.53, SD = 6.21). Another 17 subjects scoring fifty or above were assigned to the high-anxiety group (mean trait score = 57.18, SD = 4.33; mean age 26.12, SD = 5.60). Both groups consisted of only females that were matched for age and years of schooling. All participants were native speakers of German, right-handed (as assessed by the Edinburgh Handedness Inventory, Oldfield, 1971) and exhibited no current axis I disorders, as diagnosed by the Mini-International Neuropsychiatric Interview (M.I.N.I., Sheehan et al., 1998). None took part in our earlier study (Eden et al., 2014).

### Materials

Forty-four pseudowords (e.g., “muxo,” “alep”) served as learning materials, presented visually during learning and testing. (Stimulus material and result files are available from the corresponding author). All pseudowords were disyllabic and phonotactically legal (in German). They were taken from Breitenstein and Knecht (2002; Breitenstein et al., 2007), who tested the stimuli for emotional neutrality and low similarity to existing German words. The selected 44 pseudowords were randomly assigned to 44 pictures depicting concrete objects. Half displayed neutral objects such as a bucket or a chair, and the other half showed arousing-negative objects such as a gun or a shark. Pictures were color photos taken from Hemera Photo Objects, Wikipedia Commons^1^ and the International Affective Picture System (Lang et al., 1999). Some pictures were cropped to ensure that only one object was visible and positioned in the center. (See Appendix in Supplementary Material for a list of all pseudowords and matched concepts. The picture material can be requested from the author).

A pre-test assessed neutral or arousing-negative appraisal of the pictures. Thirty participants (psychology students from the University of Münster) were presented with 100 pictures (50 subjectively judged to be negative and arousing, 50 subjectively judged neutral, non-arousing). Subjects rated valence and arousal of all pictures via Self-Assessment Manikin (SAM)-scales (Bradley and Lang, 1994), ranging from one (very pleasant or low arousal) to nine (very unpleasant or high arousal). The 22 most negatively rated pictures (valence: Mean = 7.85, SD = 0.30; arousal: Mean = 5.77, SD = 0.38) differed significantly from the 22 most neutrally rated (valence: Mean = 4.76, SD = 0.21; arousal: Mean = 1.90, SD = 0.21) pictures [valence: *t*(42) = 38.908, *p* < 0.001; arousal: *t*(42) = 42.149, *p* < 0.001]. These 44 pictures served as materials in the experiment. According to the German version of CELEX-Database (Baayen et al., 1995), the frequency of object names did not differ between arousing-negative and neutral concepts, *t*(42) = –0.032, *p* = 0.975. Participants who performed the pre-test rating did not take part in the main experiment.

### Design and Procedure, Analysis

During training, the subject’s task was to decide intuitively by button-press whether a visually presented pseudoword and object (color picture) matched. Training stopped as soon as the participant reached criterion, that is, a predefined level of knowledge concerning the pseudoword-picture associations (this allowed for a balanced block-procedure in the fMRI-task; see below). Participants were not informed about the upcoming recall and valence tests and received no feedback on their responses during training. The training consisted of at least six learning passes. During each learning pass, participants were confronted with one matching “correct” and one mismatching “incorrect” pseudoword-picture pair, separated by at least one other pair. Hence, after learning pass eight, for instance, participants had heard each pseudoword sixteen times, eight times paired correctly (the same pseudoword-object combination) and eight times paired incorrectly (the pseudoword paired with eight different other objects). Note that all pseudowords used in “incorrect” pairings were “correctly” paired with other pictures. Thus, all presented pseudowords could be associated with meaning, and all pseudowords and pictures appeared equally often. There were 88 pseudoword-picture pairs per learning pass (22 correct arousing-negative, 22 correct neutral, 22 incorrect arousing-negative, 22 incorrect neutral).

The training aborted when 11 arousing-negative and 11 neutral pseudoword-picture pairs were accurately identified (hit or correct rejection) for eight times. Training continued until this criterion was reached. The criterion approach ensured equal learning for all participants. Participants were confronted with 8.26 (range: 7–12) learning passes on average. The choice for 11 pairs per valence condition and eight correct answers ensured that enough pairings were learned well enough to be detectable in explicit/implicit behavioral and imaging measures. On the other hand, we wanted to avoid a ceiling effect, to be able to investigate pseudowords whose meaning was not learned to criterion. Thus, the total number of trials was not fixed and depended on the learners’ pace of learning. Left/right assignment of “correct” and “incorrect” answers to reponse buttons was counterbalanced across participants. All participants finished within an hour (44 min on average). Presentation font size was 48, on a 15′ monitor. All stimuli were presented centered, in white against a black background. All trials began with a fixation cross (500 ms), followed by a pseudoword (1000 ms). Another fixation cross (300 ms) and a picture (1000 ms) followed. Afer 3000 ms, a red exclamation mark ended the trial, providing sufficient time for the subjects to decide whether pseudoword and pictured object matched. If no answer was given, the next trial was initiated. If a button was pressed within the 3000 ms interval, the next trial began immediately. The training and the fMRI-paradigm for this study (described below) were programmed with Presentation^®^ Software^2^ (Version 12.1, Neurobehavioral Systems, Inc., Albany, CA, USA).

Explicit knowledge of all picture-pseudoword pairings was assessed via cued-recall. Subjects were presented with the pseudowords in written format (cues) and were asked to write down the corresponding German word (comparable with a translation or vocabulary test). The cued-recall test was administered four times: directly after, 4 days after, 8 days after, and 12 days after learning. A pseudoword-valence rating assessed the transfer of valence from objects to pseudowords. Subjects were asked to spontaneously and intuitively rate the pseudowords in terms of valence, on a scale ranging from minus five (very negative) to five (very positive), with zero marked as neutral. The valence rating was administered five times: directly before, directly after, 4 days after, 8 days after, and 12 days after learning. Note that the last three cued-recall tests and the last three valence ratings were carried out online^3^, while all assessments on the first day took place in the Institute for Biomagnetism and Biosignalanalysis (Faculty of Medicine, University of Münster). Participants received written instructions but were not informed that their memory for the pseudowords would be tested.

The fMRI measurement took place in the Department of Clinical Radiology (Faculty of Medicine, University of Münster) 2 days after the training, allowing for memory consolidation through sleep (Payne and Kensinger, 2010, 2011; Bennion et al., 2013). The fMRI measurement used a block design, with six blocks, crossing valence (arousing-negative, neutral) with learning achievement (explicitly learned, less well-learned) and two additional blocks with completely novel pseudowords. Since learning outcome varied between participants (i.e., which picture-word pairs a participant had learned), the respective blocks were individually arranged for every participant. For this purpose, the pseudowords were divided into the eleven best, i.e., “explicitly learned” and the eleven remaining “less well-learned” pseudowords, individually for each valence condition. This was done on the basis of each participant’s results from the cued-recall test directly after training. The novel pseudowords, taken from the corpus of Breitenstein and Knecht (2002), had not been used during training. Each block constisted of 11 pseudowords. The presentation format was the same as during training. Each pseudoword was presented for 950 ms, with a fixed interstimulus interval of 150 ms. The six blocks (explicitly learned arousing-negative pseudowords, explicitly learned neutral pseudowords, less well-learned arousing-negative pseudowords, less well-learned neutral pseudowords, and two blocks of novel pseudowords) were presented in a pseudo-randomized order, to control for sequence effects. A 12500 ms resting phase (white fixation cross centered on a black screen) followed each block. Each block was presented twice, resulting in 26 instances in each learning condition, and 52 instances of novel pseudowords. In all, the paradigm took approximately 9 min. The stimuli were projected onto a screen at the rear end of the MR tunnel, using a beamer shielded against RF interference. Participants were instructed to read the words attentively. No further instruction was given.

### Image Acquisition

Magnetic resonance imaging scanning was performed on a 3 T whole-body scanner (Gyroscan Intera T3.0, Philips Medical Systems, Best, Netherlands) equipped with Quasar Dual gradients (nominal gradient strength in the setting used for fMRI 40 mT/m, maximal slew rate 200 mT/m/ms). For spin excitation and resonance signal acquisition, a circularly polarized transmit/receive birdcage head coil with an HF reflecting screen at the cranial end was used. T2^∗^ functional data were acquired using a single-shot echo planar (EPI) sequence (whole brain coverage, TE = 30 ms, TR = 2.5 s, FA = 90°, slice thickness 3.6 mm, interleaved acquisition order, no gap, matrix 64 × 64, FOV 230 mm, in-plane resolution 3.6 mm × 3.6 mm). The 40 transversal slices were tilted 25° from the AC/PC line in order to minimize drop out artifacts in the orbitofrontal and mediotemporal region.

### Cued-Recall Analyses

Answers in the cued-recall test (translation of pseudowords into German) were treated as correct if they described the intended object (e.g., sofa), were synonyms (e.g., couch), or subordinate-category responses that were correct descriptions of the depicted object (e.g., chesterfield). Responses were regarded incorrect if they described the superordinate category (e.g., furniture), semantically related objects (e.g., armchair), or unrelated objects (e.g., scissors). Incorrect answers and misses (no answer given) were excluded from further analyses. Means of correctly translated pseudowords were subjected to an ANOVA with the additional factor *session*, with four levels (immediately after, 4, 8, and 12 days after). This resulted in a 2 (*pseudoword affect*: arousing-negative versus neutral) × 4 (*session*) × 2 (*trait anxiety*: high versus low) mixed within/between design.

### Valence-Rating Analyses

The factor *session* had five levels in the analysis of pseudoword valence ratings: before, immediately after, 4, 8, and 12 days after training. Mean valence ratings were calculated for arousing-negatively and neutrally linked pseudowords. With a 2 (*pseudoword affect*) × 5 (*session*) × 2 (*trait anxiety*) mixed within/between design, the development of valence ratings was investigated over time.

### Image Analysis

The imaging data were analyzed with the Statistical Parametric Mapping software^4^ (SPM8, Wellcome Department of Cognitive Neurology, London, UK) implemented in Matlab (Mathworks Inc., Natick, MA, USA). Preprocessing included unwarping, realignment and normalization to the standard MNI space (Montreal Neurological Institute). Smoothing was conducted with an isotropic three-dimensional Gaussian filter with a Gaussian kernel of 6 mm full width at half maximum (FWHM). Afterward, on the first level, we applied a general linear model to the data (modeled with the canonical hemodynamic response function). The conditions were: explicitly learned arousing-negative, explicitly learned neutral, less well-learned arousing-negative, less well-learned neutral, and novel. The eight contrasts of interest were: (1) Explicitly learned pseudowords vs. novel pseudowords, (2) Less well-learned pseudowords vs. novel pseudowords, (3) Arousing-negative pseudowords vs. neutral pseudowords, (4) Explicitly learned arousing-negative pseudowords vs. less well-learned arousing-negative pseudowords, (5) Less well-learned arousing-negative pseudowords vs. less well-learned neutral pseudowords, (6) Less well-learned neutral pseudowords vs. novel pseudowords, (7) Less well-learned arousing-negative pseudowords vs. novel pseudowords. (8). Neutral pseudowords vs. novel pseudowords. These contrasts were also analyzed in a second analysis, taking into account the individual trait anxiety score as a covariate (regression analysis). Please note that due to the criterion-based design and the differentiation into explicitly learned and less well-learned words, there are to the best of our knowledge no (fMRI) studies with a comparable orientation. Thus, the contrasts investigating such differences (i.e., contrasts 2, 5, and 6) are more exploratory in nature.

To control for multiple testing on the second level (group) random-effects analysis, all group results were calculated using a combined height and extent threshold based on Monte–Carlo simulations, as implemented in the AlphaSim program (Forman et al., 1995). Based on this technique, we maintained a corrected false-positive detection rate for the amygdala, our region of interest (ROI) analysis, at *p* < 0.05, with a cluster extent (k) empirically determined by computing 1000 simulations (yielding *k* = 45 for the bilateral amygdala).

According to our hypotheses, ROI analyses of the bilateral amygdala were performed for all contrasts by one-sample *t*-tests, including all individual contrast maps of the first level. For this purpose, a mask for the bilateral amygdala was created with the aid of the WFU PickAtlas (Maldjian et al., 2003) implemented in the SPM-software. The defined mask was dilated (according to the AAL Atlas (Tzourio-Mazoyer et al., 2002) by 1 mm in radius. The regression analysis tested our *a priori* hypothesis concerning the relation between amygdala activity and degree of trait anxiety in the contrasts defined above. Voxelwise tests inside the ROI were performed and activity within the amygdalae was correlated with STAI-T (trait anxiety) scores separately for each subject.

## Results

### Cued-Recall

**FIGURE 1:**
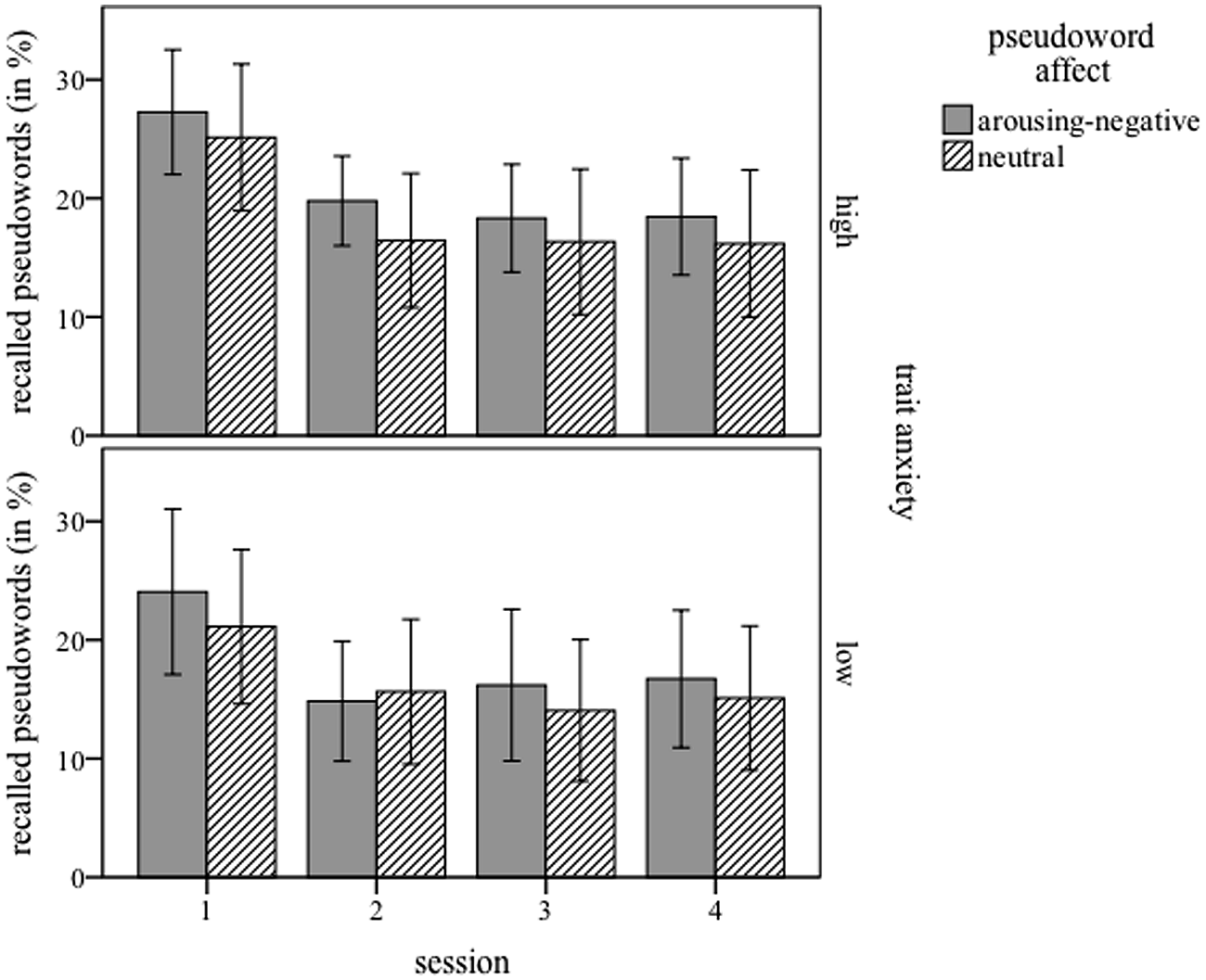
**Percentage of correct responses in the cued-recall test, for four sessions (1 = immediately, 2 = 4 days, 3 = 8 days, and 4 = 12 days after training), for high-anxiety **(Upper)** and low-anxiety **(Lower)** participants.** Pseudowords paired with arousing-negative content are presented in gray bars; neutrally linked pseudowords in dashed bars. Error bars represents 1 SE.

**Figure 1** displays the recall rates (correct translation) immediately after, 4, 8, and 12 days after training, for both participant groups and pseudoword affects. Note that performance is displayed in percentage correct, while statistical analyses were done on absolute values (maximum = 44). As expected, the brief and shallow training yielded moderate recall results. These were highest in the first session immediately after training (about 25% correct translations), decreased to around 15% correct translations by the second session 4 days later, but remained stable in session three and four (8 and 12 days after learning). The main effect of *session* was significant: *F*(3,96) = 50.655; *p* < 0.001, and is best explained by a linear effect: *F*(1,32) = 56.493; *p* < 0.001. Overall, pseudowords linked with arousing-negative pictures were recalled significantly better than neutrally linked ones, which is reflected in a main effect for *pseudoword affect, F*(1,32) = 4.347; *p* = 0.045. The three-way interaction between *pseudoword affect, session*, and *trait anxiety* also reached significance *F*(3,96) = 6.523; *p* = 0.013.

Additional ANOVAS (*word affect* × *session*), separately for each group, further investigated this interaction. The data of the low-anxious group showed a main effect of *session F*(3,48) = 22.690; *p* < 0.001, and the interaction *pseudoword affect* × *session, F*(3,48) = 8.843; *p* = 0.005. The main effect of *pseudoword affect* did not reach significance *F*(1,16) = 1.652; *p* = 0.217. *Post hoc t*-tests calculated to assess the interaction yielded a significant difference between arousing-negative (Mean = 11.06, SD = 5.910) and neutral pseudowords (Mean = 9.29, SD = 5.565) at session one [*t*(16) = 2.624; *p* = 0.018]. No other session showed such a difference (all *p* > 1.444). The data for the high-anxious group also yielded a main effect of *session*: *F*(3,48) = 28.448, *p* < 0.001. No other main effects or interactions reached significance [*word affect*: *F*(1,16) = 2.698; *p* = 0.120 and *word affect* × *session*: *F*(3,48) = 0.255; *p* = 0.857].

### Pseudoword Valence Rating

**FIGURE 2:**
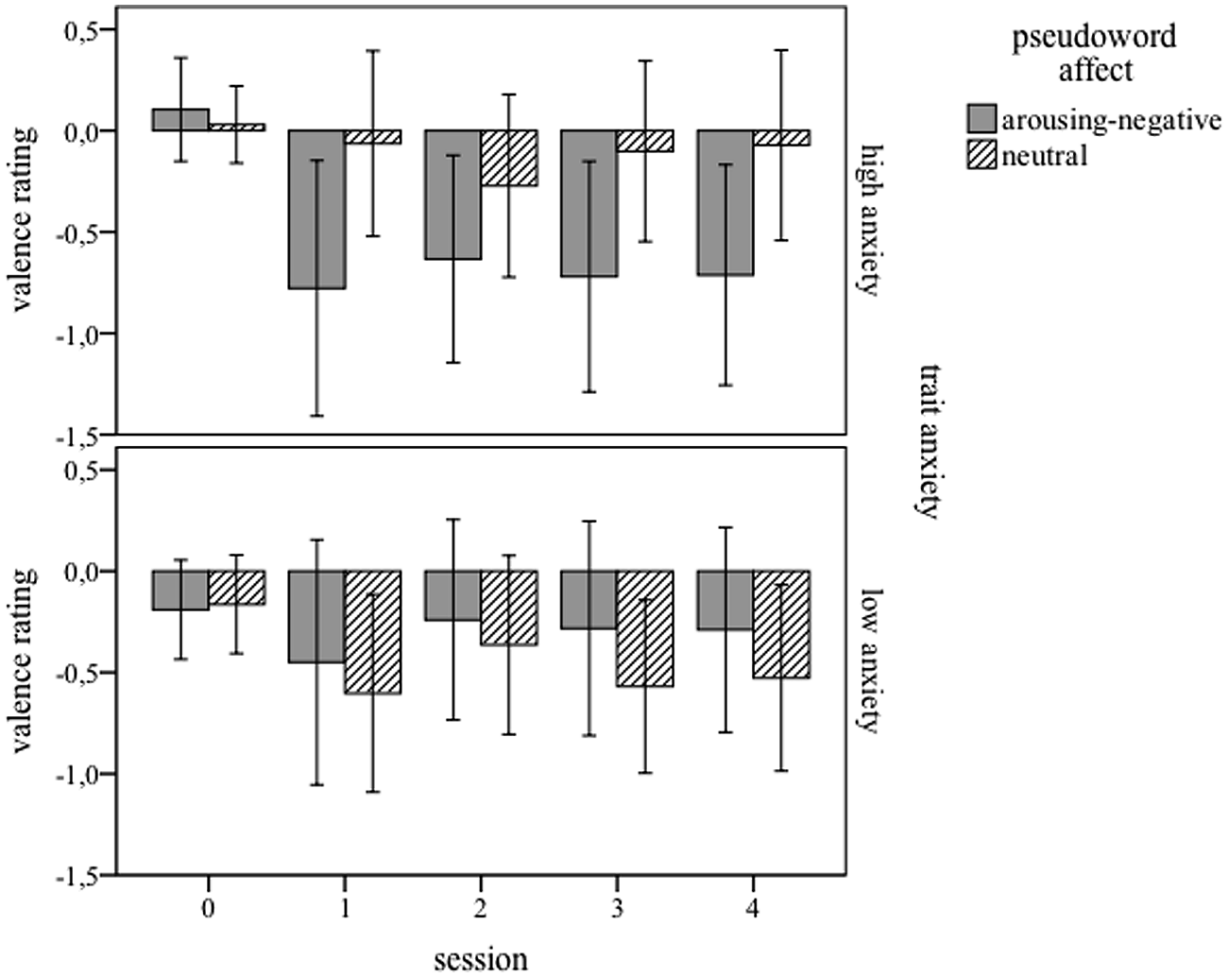
**Mean valence ratings for five sessions (0 = before training, 1 = immediately, 2 = 4 days, 3 = 8 days, and 4 = 12 days after training), and for high-anxiety **(Upper)** and low-anxiety **(Lower)** participants.** Pseudowords paired with arousing-negative content are presented in gray bars; neutrally linked pseudowords in dashed bars. Error bars represents 1 SE.

**Figure 2** displays the mean valence ratings separately for participant groups and pseudoword affect, in all five sessions. The ANOVA with *session, pseudoword affect*, and *trait anxiety* yielded no main effect of *pseudoword affect, F*(1,32) = 1.872; *p* = 0.506. The rating behavior toward a more negative rating changed significantly over time, indicated by a main effect of *session F*(4,128) = 10.335; *p* < 0.001, best described as a linear trend, *F*(1,32) = 7,823; *p* = 0.009. Although the means suggest a difference between negative and neutral pseudowords for the high-anxiety group, no other main effects or interactions reached significance. (Note: An ANOVA on the same valence data where ratings of the first session were subtracted from ratings given at the second and third session (baseline correction), yielded qualitatively the same results.)

### fMRI Results

#### Region of Interest Analysis Regarding Amygdala Responsiveness to Pseudowords

With contrasts one and two, we investigated a general learning effect. As expected, in contrast 1, explicit pseudowords elicited more amygdala activity than novel pseudowords, bilaterally *x* = 31, *y* = -10, *z* = -14, *t*(33) = 2.26, *k* = 56 voxels, *p* = 0.015 corrected. Contrast 2 was not significant, showing similar amygdala activity for less well-learned pseudowords and novel pseudowords. Contrast 3 tested for effects of pseudoword affect, independent of learning success. As expected, arousing-negative pseudowords elicited more amygdala activity than neutrally linked pseudowords *x* = -22, *y* = -8, *z* = -9, *t*(33) = 3.19, *k* = 108, *p* = 0.002 corrected. Contrast 4 tested for effects of explicit learning. There was no difference in amygdala activity between explicitly learned and less well-learned arousing-negative pseudowords. With contrasts five to seven, effects of acquired affect were investigated for pseudowords that are not well learned or remembered. As expected (and in line with contrast 3), contrast 5 showed that arousing-negative pseudowords elicited more bilateral amygdala reactivity than neutral pseudowords *x* = -30, *y* = 4, *z* = -14, *t*(33) = 2.45, *k* = 71, *p* = 0.010. However, contrast 6 (less well-learned neutral pseudowords vs. novel words), contrast 7 (less well-learned arousing-negative pseudowords vs. novel pseudowords) and contrast 8 (neutral pseudowords vs. novel pseudowords) did not yield significant results. (See **Table 1** for a clear arrangement of all results above).

**Table 1:** Region of interest analysis regarding amygdala responsiveness to pseudowords.

	Contrast	T	MNI-coordinates			Cluster (*k*)	*p*
			*x*	*y*	*z*		
1	Explicitly learned pseudowords vs. novel pseudowords	2.26	31	-10	-14	56	0.015
2	Less well-learned pseudowords vs. novel pseudowords	-	-	-	-	No significant clusters	–
3	Arousing-negative pseudowords vs. neutral pseudowords	3.19	-22	-8	-9	108	0.002
4	Explicitly learned arousing-negative pseudowords vs. less well-learned arousing-negative pseudowords	-	-	-	-	No significant clusters	–
5	Less well-learned arousing-negative pseudowords vs. less well-learned neutral pseudowords	2.45	-30	4	-14	71	0.010
6	Less well-learned neutral words vs. novel words	-	-	-	-	No significant clusters	-
7	Less well-learned arousing-negative vs. novel words	-	-	-	-	No significant clusters	-
8	Neutral words vs. novel words	2.43	38	4	-23	8 (cluster below AlphaSim correction level)	0.010

*Conducted at *p* < 0.05, uncorrected (corrected at *p* < 0.05 on the cluster level using the AlphaSim procedure, which resulted in an empirically determined cluster-extent threshold of *k* = 45 voxels). Coordinates are given in MNI space.*

#### Trait Anxiety as Covariate

**FIGURE 3:**
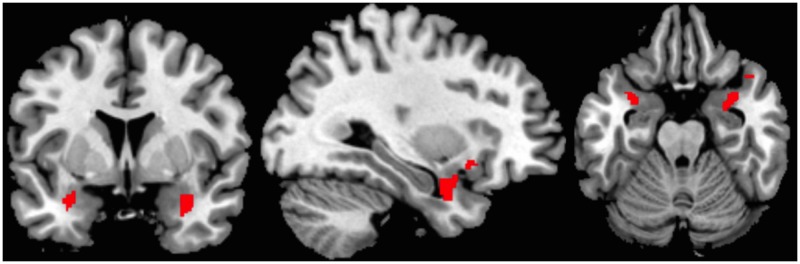
**Coronal, sagittal and axial view depicting activation extension of one example contrast; supra-threshold voxels are shown in red.** Region of interest (ROI)-analysis with trait anxiety as a covariate for the bilateral amygdala; contrast 1: Explicitly learned pseudowords > novel pseudo words (*x* = 32, *y* = 5, *z* = -21).

The ROI-analysis of the bilateral amygdala with trait anxiety as covariate revealed a significant contrast 1 (explicitly learned pseudowords vs. novel pseudowords, see **Figure 3**). Explicitly learned pseudowords elicited more amygdala activity than novel pseudowords in the bilateral amygdala, and this effect was positively related to measures of trait anxiety *x* = 32, *y* = 5, *z* = -21, *t*(32) = 2.62, *k* = 111 voxels, *p* = 0.007. Importantly, and contrary to the analysis without covariate, contrast 2 (less well-learned pseudowords vs. novel pseudowords) also yielded significant results *x* = 31, *y* = 2, *z* = -19, *t*(32) = 2.42, *k* = 77 voxels, *p* = 0.011, positively related to trait anxiety. Contrast 3 (arousing-negative pseudowords vs. neutral pseudowords) was also significant *x* = 34, *y* = -7, *z* = -11, *t*(32) = 2.74, *k* = 55 voxels, *p* = 0.005. Hence, arousing-negative pseudowords elicited more amygdala activity than neutral pseudowords, and this was positively related to trait anxiety. As in the analysis without covariate, contrast 4 (explicitly learned arousing pseudowords vs. less well-earned arousing pseudowords) was not significant. Contrast 5, comparing arousing-negative and neutral pseudowords that were not learned well, was significant *x* = 32, *y* = -7, *z* = -9, *t*(32) = 2.16, *k* = 47 voxels, *p* = 0.019. The arousing-negative pseudowords elicited more amygdala reactivity than the neutral pseudowords, again, positively related to measures of trait anxiety. Contrast 6 (less well-learned neutral pseudowords vs. novel pseudowords), however, was not significant. But contrast 7 (less well-learned arousing-negative pseudowords vs. novel pseudowords) and contrast 8 (neutral pseudowords vs. novel pseudowords), were highly significant (contrast 7: *x* = 31, *y* = 0, *z* = -23, *t*(33) = 3.25, *k* = 207 voxels, *p* < 0.001; contrast 8: *x* = 31, *y* = 4, *z* = -19, *t*(32) = 3.39, *k* = 116 voxels, *p* < 0.001. Hence, pseudowords that were less well learned elicited more amygdala reactivity than novel pseudowords, independent of the linked affect. This was positively related to measures of trait anxiety. See **Table 2** for an overview of all regression analysis results and **Figure 3** for a visualization of the spatial extension of amygdala activation of contrast 1.

**Table 2:** Regression analysis with trait anxiety as covariate.

	Contrast	T	MNI-coordinates			Cluster (*k*)	*p*
			*x*	*y*	*z*		
1	Explicitly learned pseudowords vs. novel pseudowords	2.62	32	5	-21	111	0.007
2	Less well-learned pseudowords vs. novel pseudowords	2.42	31	2	-19	77	0.011
3	Arousing-negative pseudowords vs. neutral pseudowords	2.74	34	-7	-11	55	0.005
4	Explicitly learned arousing-negativepseudowords vs. less well-learned arousing-negativepseudowords	-	-	-	-	No significantclusters	-
5	Less well-learned arousing-negative pseudowords vs. less well-learned neutral pseudowords	2.16	32	-7	-9	47	0.019
6	Less well-learned neutral words vs. novel words	-	-	-	-	-	-
7	Less well-learned arousing-negative vs. novel words	3.25	31	0	-23	207	<0.001
8	Neutral words vs. novel words	3.39	31	4	-19	116	<0.001

*Conducted at *p* < 0.05, uncorrected (corrected at *p* < 0.05 on the cluster level using the AlphaSim procedure, which resulted in an empirically determined cluster-extent threshold of *k* = 45 voxels). Coordinates are given in MNI space.*

Note that the significant contrasts showed cluster within the amygdala’s VOIs after correction for multiple testing (see **Tables 1** and **2**). Subnuclei of the amygdala could not be distinguished by our methods.

## Discussion

We analyzed the development of a memory bias for novel and neutral stimuli before and after a learning phase, during which the pseudowords were paired with pictures with arousing-negative or neutral content. High and low trait anxious persons participated. Our results demonstrate that a brief training in an associative learning paradigm suffices to elicit a memory bias for those pseudowords that were combined with arousing, negative pictures, compared to those that were linked to neutral pictures. This bias became evident in behavioral as well as in fMRI measures. A cued-recall translation test, administered directly after and at four 4-day-intervals following training, showed better recall for pseudowords that had been combined with arousing-negative content. However, the valence ratings showed no differences between arousing-negative and neutral pseudowords. The fMRI measurement that took place 2 days after learning showed a hyperactivation of the amygdala in response to the arousing-negative pseudowords, indicating that these stimuli were processed differently from neutrally linked pseudowords. Most interestingly, this hyperactivation was more pronounced in high-anxious persons, replicating previous behavioral results (Eden et al., 2014). The effect was not restricted to pseudowords that participants could explicitly remember. In fact, it seems that the amygdala of high trait-anxious persons reacted sensitively to all stimulus material, an indication for generalization. Note, however, that the behavioral data showed no general enhancement for negative-arousing over neutral pseudowords for high-anxious individulas, not even in session one, where we saw an effect for low-anxious persons. Moreover, and against our expectation, there were no significant changes in memory bias from session two onward. The latter two findings stand in contrast to our earlier study (Eden et al., 2014). We will discuss each of these aspects in turn.

As expected, very few (i.e., six to nine) correct and incorrect pairings in an associative learning paradigm resulted in a memory bias for pseudowords that were paired with negative-arousing pictures. This, once again, shows the effectiveness of this paradigm for word learning (Dobel et al., 2009, 2010; Eden et al., 2014). After learning participants showed a memory bias in the cued-recall test. Thus, all participants, independent of their level of trait anxiety, were better able to translate pseudowords that had been combined with arousing-negative content than pseudowords linked with neutral content. This replicates earlier findings, where participants were better able to memorize stimuli with aversive emotional content (e.g., Mitte, 2008). Here, as in our earlier work (Eden et al., 2014), we observe this advantage even after a very brief associative training. However, the same effect was not found in the (implicit) valence rating. Participants rated arousing-negative pseudowords more negative than neutral ones, but this difference did not reach significance (see **Figure 2**). Thus, participants showed an explicit but no implicit memory bias. This is in line with the results of a meta-analysis by Mitte (2008), who indeed showed that implicit memory effects are seldom found. Some authors even question the existence of an implicit memory bias (e.g., McCabe, 1999; Russo et al., 1999). An explanation why explicit tests yield the memory bias but the implicit ones do not, could be as follows. According to Scott et al. (2009), valence features are part of the semantic representation of words. Hence it can be assumed that the activation of such features is required in explicit tasks such as cued-recall. In this task, participants had to perform a one-to-one mapping of a pseudoword to an existing German word. In contrast, an implicit task such as the valence rating applied here does not require the activation of German words, with their semantic and valence features. This may explain why implicit memory bias effects for words are so much harder to detect than explicit ones. Note that we did obtain implict bias effects in our earlier study (Eden et al., 2014), which had more power in terms of items and partipants. The missing effect in the valence rating might at first glance invite to speculate about the processing depth of learned words and seems to suggest a shallow encoding. However, results of former studies from our group that used very similar associative word-learning paradigms (e.g., Breitenstein et al., 2007; Dobel et al., 2009, 2010; Liuzzi et al., 2010) strongly suggest that meaning is indeed acquired for the pseudowords. Breitenstein et al. (2007) showed with crossmodal priming that learned pseudowords primed existing words related to their acquired meaning as effectively as native-language words. This was corroborated by Dobel et al. (2010), who applied magnetencephalography (MEG). They showed that the N400 component [an indicator for semantic (mis)matches between word and picture] to pictures was strongly reduced when they were preceded by pseudowords whose acquired meaning corresponded to the pictured concept. Based on these and other findings, we feel confident that the learning effects found in the present study truly reflect semantic/emotional learning, and that the pseudowords do not simply present superficial mnemonic cues to existing words and/or corresponding pictures.

We now turn to the fMRI data, for which we carried out two analyses: the first analysis served to investigate the general effect of memory bias, and the second to additionally assess the influence of trait anxiety. With respect to the first analysis, we observed general effects of learning. Explicitly learned pseudowords elicited more amygdala activity than novel pseudowords, showing that only few repetitions of pseudoword-picture pairs sufficed to activate the amygdala, even 2 days after learning. This amygdalar hyperactivation was seen for explicitly learned pseudowords only, not for the set of “not so well learned” pseudowords (the worst 11 for each participant). This suggests that they either were not learned at all, or that their superficial learning history leads to different processing or storage – evident in explicit recall and amygdala reactivity. The third contrast investigated pseudoword affect, more precisely whether arousing-negative pseudowords generally elicited more amygdala activity than neutral ones. Importantly, this was the case, when looking for explicitly and less well learned pseudowords together. This effect corroborates the memory bias found in prior studies (for a review, see Mitte, 2008) and the main effect in the cued-recall data of the current study. Of interest is whether this neural correlate for a memory bias is driven solely by the explicitly recalled items. This, as contrast 4 showed, was not the case: Explicitly learned and less well-learned arousing-negative pseudowords elicited equal amygdala reactivity. This clearly goes against the suggestion that nothing is learned when stimuli cannot be recalled explicitly. Clearly, aversive stimuli have similar amygdalar effects, independent of their level of explicit recall. To explore this further, we contrasted less well-learned arousing-negative and neutral pseudowords (contrast 5), and observed more amygdala reactivity for the arousing-negative stimuli. This corroborates the above finding that affect is indeed acquired for these pseudowords, even though participants could not explicitly translate these words very well. These findings corroborate the observed amygdala sensitivity for emotionally arousing stimuli – even in the absence of explicit memory (e.g., Dannlowski et al., 2007a,b; Pichon et al., 2012; Suslow et al., 2013). Note, however, that the overall amygdala activation of these less well-learned pseudowords did not differ from the activation for completely novel pseudowords. This contrast was thus only significant for explicitly learned stimuli. There is ample evidence that completely novel stimuli are processed differently from items that have been seen before. Repetition decreases amygdala activity, and only explicitly learned and recalled stimuli overcome this repetition suppression (e.g., Ishai et al., 2004; Wendt et al., 2011). Finally, contrast 8 was implemented to investigate effects of generalization, comparing all neutrally linked pseudowords (explicitly and less well-learned) with all novel pseudowords. Both conditions elicited about equal amygdala activity. This provides no evidence for generalization. Neutral words were not associated or confounded with negative affect, and that the hyperactivation of the amygdala for arousing-negative pseudowords was indeed due to this negative affect.

The second fMRI-analysis, with the factor trait anxiety as a covariate, revealed the following. As expected and as in the first fMRI-analysis, explicitly learned pseudowords elicited more amygdala reactivity than novel pseudowords in the bilateral amygdala, and this effect was positively related to measures of trait anxiety. The effect increases with increasing levels of trait anxiety, showing that persons with higher levels of trait anxiety process stimuli, gathered in emotionally arousing situations, differently from persons with low levels of anxiety (e.g., Tolkunov et al., 2010; Asakawa et al., 2014; Burgess et al., 2014). Different from analysis one, the contrast was also significant for less well-learned pseudowords vs. novel pseudowords. This lends support to the amygdala’s sensitivity (i.e., hyperreactivity) in persons with high levels of trait anxiety. Contrast 3 investigated the occurrence of a memory bias. As expected and as in analysis one, the arousing-negatively linked pseudowords elicited more amygdala activity than the neutrally linked pseudowords, supporting similar prior studies (Laeger et al., 2012, 2014a,b). Contrast 4, comparing the explicitly and less well-learned arousing-negative pseudowords, was – again – not significant. Thus, even when taking into account the trait anxiety levels, the amygdalae of our participants did not differentiate between explicitly and less well-learned pseudowords when both had been combined with negative content. Contrast 5 again corroborated that even the less well-learned arousing-negative pseudwords elicited more amygdala reactivity than less well-learned neutral pseudowords. In addition, this effect is the more pronounced the higher the level of trait anxiety. This shows that our high trait-anxious participants processed the arousing-negative words differently from the neutral words, although they could not translate these pseudowords very well. As in the first analysis, contrast 6 showed no differences between less well-learned neutrally linked pseudowords and completely novel pseudowords. Differently from analysis one, contrast 7 did reveal an effect. Less well-learned arousing-negative pseudowords elicited stronger amygdala reactivity than novel pseudowords, and the effect was stronger the higher the level of trait anxiety. This finding, once again, supports the sensitivity of trait-anxious persons for emotionally aversive stimuli, even if distinct explicit memory for these is not present. Contrast 8, investigating potential generalization effects, turned out to be significant. In contrast to analysis one, neutral pseudowords elicited more amygdala activity than novel pseudwords, and this effect was stronger with higher levels of trait anxiety. We believe that this is especially interesting, since it shows the down side of sensitivity in situations with aversive content. This sensitivity, that is, the hyperactivation of amygdalae in response to aversive situations/stimuli, may have evolved from evolutionary mechanisms, to protect humans from getting killed in dangerous situations. However, the final contrast shows that the amygdala of highly trait anxious persons overreacts in response to neutrally linked stimuli. This is an effect of generalization, found in earlier studies (e.g., Eden et al., 2014; see Resnik and Paz, 2014 for an animal model of the underlying mechanisms of generalization) and probably results from a transfer of aversion from arousing-negative stimuli to neutral stimuli during learning.

In the current study, we tried to overcome some criticism to earlier studies. Behavioral testing was done at various points after learning, to assess consolidation over time. We combined behavioral and imaging measures and differentiated between explicit and more implicit (“less well-learned”) items. We showed that the behavioral data do not change much from the second measurement onward. Explicit recall is better, overall, for negatively paired stimuli, but this effect is not significant in high-anxious individuals. The fMRI measures revealed an interesting pattern of results, with (1) more amygdala activation for explicitly learned than for novel stimuli; (2) evidence for memory bias, with more activation for pseudowords that were combined with negative content than for those paired with neutral content, (3) evidence that this memory bias was independent of the explicit learning success, but (4) dependent on trait-anxiety measures and (5) a difference in amygdalar activation between less well-learned pseudowords and completely novel ones that depended on trait-anxiety measures.

We would like to point out some limitations, caveats and open questions that should be addressed in future studies. First, we investigated two extreme groups. This well-established approach does not allow making predictions or drawing conclusions about the memory bias in persons with moderate levels of trait anxiety. Thus, we recommend that future studies integrate a third group of persons with moderate anxiety levels, or use a design that takes individual (trait) anxiety scores into account, as was done in the analysis of our fMRI data. Second, although we controlled for individual learning histories concerning the items relevant for behavioral and fMRI analyses, by use of valence-free pseudowords, we could not control individual differences concerning the pictures used to link negative-aversive or neutral valence to the pseudowords. Hence, despite the pre-test, the pictures might have evoked varying emotions to varying degrees in our participants, which results in unknown variance in acquired emotionality of the pseudowords. This problem is common to all studies that use neutral and aversive stimuli, and an assessment of the stimuli by the study participants themselves may be of help. Third, in the behavioral part of this study we used similar stimuli and measurements as in our earlier study (Eden et al., 2014), but did not exactly replicate the results. In Eden et al. (2014), participants rated all arousing-negative stimuli more negative than neutral ones, and ratings differed between high- and low anxious individuals. Differences between the studies concern the number of items (44 instead of 60) and participants (34 instead of 54), and repetitions during training (7–12; i.e., 8.25 on average instead of 5). In both studies, recall is better for negative than for neutral pseudowords, but the interaction with participant group shows a different pattern. Power differences might be responsible for these differences. Next, in contrast to our earlier study, we observed no significant differences between neutrally and negatively paired stimuli in the valence ratings of high-anxious individuals. Again, the patterns are similar but there is less power in the current study. The differing results in two similar valence ratings actually stress that the implicit memory bias is indeed hard to replicate and not robust (for a meta-analytic review, see Mitte, 2008). Fourth, there is generally a positive correlation between (trait) anxiety and depression. Both anxiety and depression have been associated with a failure to adequately regulate the amygdala via top–down mechanisms (Johnstone et al., 2007). Thus, effects reported in anxiety research might partly be due to a potential depression, and vice versa. This is the reason why we used the reliable and well-validated M.I.N.I interview into our study, ensuring that none of our participants (ever) suffered from an affective disorder. However, the M.I.N.I is a dichotomous tool (a disorder is present or not). We thus cannot rule out the existence of subclinical depression, although none of our participants showed any signs. We suggest that future studies additionally apply a continuous measurement that reveals the intensity of a potential subclinical depression [such as the Beck Depression Inventory (BDI) or the Hamilton Depression Scale (HAMD), Hamilton, 1960; Beck et al., 1961]. With such measures, potential effects can be more clearly attributed to affective or anxiety disorders. Furthermore, the current study only focuses on the negativity bias and neglects the so-called positivity bias, a self-serving attribution bias that represents a well-attested and robust phenomenon in human cognition (e.g., Bradley, 1978; Zuckerman, 1979; Campbell and Sedikides, 1999). In their meta-analytic review, Mezulis et al. (2004) investigated numerous samples and showed that the bias was smallest for anxiety and depression patients. Since the sample of our study consists of highly trait anxious (potentially small positivity bias) and highly non-anxious persons (potentially large positive bias), it would be an interesting research issue to assess the extent of this bias and the difference between the two groups. However, this was not the aim of our study. We followed a strict hypothesis-driven approach and compared only negative arousing and neutral stimuli. Future studies might include positive arousing stimuli into the paradigm. This would of course lengthen the learning phase, and might thus considerably change the implicit results. Besides, the current study did not investigate mood-congruency effects. Mood congruency describes the phenomenon that emotional information congruent with the current mood is more likely to be recalled than information that is incongruent with the current mood (Bower, 1981). In many studies it has been shown that mood-congruent depressive information is likely to be recalled by persons in a depressed mood (for a meta-analytic study on explicit recall, see Matt et al., 1992; for a meta-analytic study on implicit recall, see Gaddy and Ingram, 2014). Patients suffering from major depressive disorder exhibited preferential recall of negative stimuli, dysphoric persons did showed no preferred recall of negative or positive stimuli, and healthy controls tended to recall positive stimuli. With the current design, we cannot decide whether non-clinical mood congruency processing played a role, because we did not implement a mood measurement. Given that anxiety and depression are highly correlated and in the absence of mood information, we cannot rule out that a depressive, an anxious or any other kind of negative mood is partially responsible for the obtained effects. A second kind of mood congruency (a congruency between behavior/symptoms and mental disorders) is certainly at stake in the study at hand. The people in our sample showed no clinical symptoms, but since trait anxious persons exhibit a greater risk to develop anxiety disorders, it is very likely that the effect found in our study can be traced back to this “anxious mood.” Furthermore, we performed an fMRI measurement with a blocked design, because it is sensitive to small effects. Another improvement of future studies would be the use of an event-related design.

Finally, it is yet unclear how emotion and feelings are implemented and operated at the level of words, and how emotional information conveyed by words modulates and regulates emotional experience. These questions are currently under debate (see several contributions to this Frontiers research topic). However, what we do know and were able to show here is that persons with high levels of trait anxiety exhibit dysfunctional learning and memory mechanisms for affective verbal stimuli. This might originate from evolutionary shaped, adaptive behavior that maximizes chances of survival due to withdrawal from potentially threatening situations. However, with the tremendous changes in many modern societies during the last centuries, the advantage of this sensitivity diminishes continuously. Today, highly anxious persons, who possess this sensitivity as a character trait, primarily suffer from a higher probability to develop anxiety disorders, the most prevalent class of all psychological disorders (e.g., Kessler et al., 2005). As suggested by others (e.g., Lissek, 2012; Resnik and Paz, 2014), neural hyperactivity to items with only brief learning histories (that are explicitly not well remembered), together with generalization, might be underlying mechanisms in the development of anxiety disorders. As we hope to have illustrated here, learning of emotional words constitutes an important and experimentally well-controlled approach to investigate this further. To support individual health and to prevent high burden on health care systems, it is crucial to better understand the processes and mechanism that underlie the development of anxiety disorders, and to identify persons at risk.

## Conflict of Interest Statement

The authors declare that the research was conducted in the absence of any commercial or financial relationships that could be construed as a potential conflict of interest.

## Footnotes

1 http://commons.wikimedia.org

2 www.neurobs.com

3 http://www.limesurvey.org/

4 www.fil.ion.ucl.ac.uk/spm
