# Supplementary material for: Brief learning induces a memory bias for arousing-negative words: an fMRI study in high and low trait anxious persons
Source: Front Psychol. 2015 Aug 21;6:1226. doi: 10.3389/fpsyg.2015.01226 (PMC4543815; doi:10.3389/fpsyg.2015.01226)
Supplement: Supplementary file 1 [file Data_Sheet_1.DOCX]

Appendix

Applied pseudowords with matched concepts (as presented in German with corresponding English translations) for conditions A and B:

| Condition A | | Condition B | |
| --- | --- | --- | --- |
| bans | Foltergerät (torture tool) | lilt | Foltergerät |
| deme | Elektrischer Stuhl (electric chair) | kela | Elektrischer Stuhl |
| ifro | Brandopfer (fire victim) | aleo | Brandopfer |
| molb | Hai (shark) | nolo | Hai |
| rida | Unfall (accident) | gohg | Unfall |
| pird | Darm (intestine) | pari | Darm |
| kage | Kotze (puke) | maku | Kotze |
| binu | Kadaver (cadaver) | olor | Kadaver |
| upan | Hakenkreuz (swastika) | tihe | Hakenkreuz |
| ralm | Kindersoldat (child soldier) | efes | Kindersoldat |
| kurn | Skinhead (skinhead) | unam | Skinhead |
| lasu | Akne (acne) | inwa | Akne |
| bila | Massengrab (mass grave) | take | Massengrab |
| pods | Legebatterie (laying battery) | ured | Legebatterie |
| nara | Tumor (tumour) | agep | Tumor |
| agod | Eiter (purulence) | apef | Eiter |
| digu | Wunde (wound) | gult | Wunde |
| nalf | Schlange (snake) | enik | Schlange |
| azan | Terrorist (terrorist) | silg | Terrorist |
| romp | Neonazi (neo-nazi) | atry | Neonazi |
| bahe | Flutwelle (tsunami) | enos | Flutwelle |
| gano | Guillotine (guillotine) | glum | Guillotine |
| lilt | Walnuss (walnut) | bans | Walnuss |
| kela | Bürste (brush) | deme | Bürste |
| aleo | Pfanne (pan) | ifro | Pfanne |
| nolo | Lupe (magnifier) | molb | Lupe |
| gohg | Lineal (ruler) | rida | Lineal |
| pari | Gabel (fork) | pird | Gabel |
| maku | Steckdose (socket) | kage | Steckdose |
| olor | Regenschirm (umbrella | binu | Regenschirm |
| tihe | Lampe (lamp) | upan | Lampe |
| efes | Knopf (button) | ralm | Knopf |
| unam | Schraube (screw) | kurn | Schraube |
| inwa | Schlüssel (key) | lasu | Schlüssel |
| take | Zahnbürste (toothbrush) | bila | Zahnbürste |
| ured | Toaster (toaster) | pods | Toaster |
| agep | Schere (scissors) | nara | Schere |
| apef | Tastatur (keypad) | agod | Tastatur |
| gult | Lastwagen (truck) | digu | Lastwagen |
| enik | Kompass (compass) | nalf | Kompass |
| silg | Toilettenpapier (toilet paper) | azan | Toilettenpapier |
| atry | Kerzenständer (candleholder) | romp | Kerzenständer |
| enos | Gürtel (belt) | bahe | Gürtel |
| glum | Kamm (comb) | gano | Kamm |
